# A spectroscopic hike in the U–O phase diagram

**DOI:** 10.1107/S1600577521010572

**Published:** 2021-11-03

**Authors:** Damien Prieur, Marie-Margaux Desagulier, Daniel R. Neuville, Christine Guéneau, Enrica Epifano, Kathy Dardenne, Joerg Rothe, Philippe Martin

**Affiliations:** a Helmholtz Zentrum Dresden-Rossendorf, Institute of Resource Ecology, PO Box 510119, 013014 Dresden, Germany; b The Rossendorf Beamline at ESRF – The European Synchrotron, CS40220, 38043 Grenoble Cedex 9, France; c CEA, DES, ISEC, DMRC, University of Montpellier, Marcoule, France; d Université de Paris, Institut de Physique du Globe de Paris, CNRS, 75238 Paris Cedex 05, France; e Université Paris-Saclay, CEA, Service de la Corrosion et du Comportement des Matériaux dans leur Environnement (SCCME), 91191 Gif-sur-Yvette, France; f CIRIMAT Laboratory, University of Toulouse, CNRS, INPT, UPS, ENSIACET, 4 Allée Emile Monso, BP-44362, 31030 Toulouse Cedex 4, France; g Karlsruhe Institute of Technology (KIT), Institute for Nuclear Waste Disposal (INE), PO Box 3640, D-76021 Karlsruhe, Germany

**Keywords:** UO_2_, *in situ* XANES, thermodynamics, CALPHAD method

## Abstract

The U–O phase diagram is of paramount interest for nuclear-related applications and has therefore been extensively studied. Experimental data have been gathered to feed the thermodynamic calculations and achieve an optimization of the U–O system modelling. Although considered as well established, a critical assessment of this large body of experimental data is necessary, especially in light of the recent development of new techniques applicable to actinide materials. Here we show how *in situ* X-ray absorption near-edge structure (XANES) is suitable and relevant for phase diagram determination. New experimental data points have been collected using this method and discussed in regard to the available data. Comparing our experimental data with thermodynamic calculations, we observe that the current version of the U–O phase diagram misses some experimental data in specific domains. This lack of experimental data generates inaccuracy in the model, which can be overcome using *in situ* XANES. Indeed, as shown in the paper, this method is suitable for collecting experimental data in non-ambient conditions and for multiphasic systems.

## Abstract 2

*In situ* XANES is a powerful method to collect new experimental data in the U–O phase diagram.

## 1. Introduction

As for any element of the periodical table, actinide oxides chemistry, physics, thermodynamics and material science depend on the oxidation states. In the actinide series, uranium is of foremost importance in regard to its technological significance in nuclear-related applications (Burns *et al.*, 2012 ▸). Among the variety of stoichiometric and non-stoichiometric U oxide phases, uranium exists in the +III, +IV, +V and +VI oxidation states (Kvashnina *et al.*, 2013 ▸). One of the key scientific challenges is a precise determination of uranium valence state (multiple or not) as it dictates the oxides behaviour: from their irradiation in nuclear reactor to their disposal in dedicated waste repositories (fuel thermo-physical properties, chemical reactivity in the environment, *etc*.). Thanks to its element-specificity and local bonding-sensitivity, X-ray absorption near-edge structure (XANES) is a well recognized method to assess the valence of almost any elements (Denecke, 2015 ▸; Shi *et al.*, 2014 ▸; Rothe *et al.*, 2012 ▸). Applying this synchrotron technique at room temperature has become quite standard for some of the actinides (Th, U, Pu and Am) in several dedicated beamlines (Kvashnina *et al.*, 2013 ▸; Rothe *et al.*, 2012 ▸; Fortner *et al.*, 2006 ▸; Denecke, 2016 ▸; Scheinost *et al.*, 2021 ▸). However, some of the aforementioned processes are occurring in chemical (pH, *etc*.) or thermodynamical (temperature, oxygen partial pressure, *etc*.) conditions which may vary from the ambient ones and would require then an *in situ* determination of the actinide oxidation state(s). Unfortunately, such *in situ* studies remain very scarce for reasons of the safety issues associated with the handling of radionuclides-bearing samples in non-ambient conditions. In this context, the first goal of this paper is to show both feasibility and suitability of *in situ* *L*_III_ XANES applied to uranium oxides.

**Figure 1:**
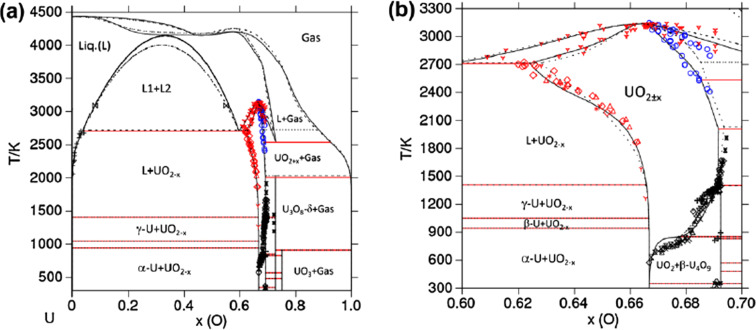
U–O phase diagram calculated using the CALPHAD modelling by Guéneau *et al.* (2011 ▸) (solid line) with the thermodynamic database TAF-ID (Guéneau *et al.*, 2021 ▸) in the whole O range (*a*) and restrained to 60 and 75 at% O (*b*). Experimental data (red, black and blue points) and associated references are detailed by Baichi *et al.* (Baichi *et al.*, 2006*a* ▸,*b* ▸; Baichi, 2001 ▸), Labroche *et al.* (Labroche, 2000 ▸; Labroche *et al.*, 2003*a* ▸,*b* ▸) and Manara *et al.* (2005 ▸). Reprinted from Guéneau *et al.* (2011 ▸), Copyright (2011), with permission from Elsevier.

As a representative and comprehensive example, this article will present our spectroscopic hike into the U–O phase diagram. This system has been extensively studied in the past decades and the current version of its phase diagram is provided in Fig. 1 ▸ (Guéneau *et al.*, 2011 ▸). The experimental data, on which are based the thermochemical modelling, are also given. As noted in the phase diagram, the stable oxide phases are UO_2±*x*_, U_4_O_9_, U_3_O_7_, U_3_O_8_ and UO_3_.

Relative to the UO_2±*x*_ fuel, the most critical parameter to assess is the deviation from stoichiometry which is noted ‘*x*’ and is specifically the gap from an O/U ratio equal to 2.00. In the U–O phase diagram, most of the O/U ratio has been derived from either X-ray diffraction (XRD) or thermogravimetric analysis (TGA). Contrary to XRD, TGA measures directly the oxygen content variation through the sample mass loss. However, accurate measurements require knowing either the initial or the final O/*M* of the studied compounds. In the case of XRD, the oxygen stoichiometry is indirectly derived from the lattice parameter using empirical relations (Ohmichi *et al.*, 1981 ▸). This methodology is generally wrongly used in the UO_2_–U_4_O_9_ domain where two oxide phases coexist (Elorrieta *et al.*, 2016 ▸). Another drawback of XRD for this type of study lies in its limited resolution associated with the respective U and O masses: the formation of higher U oxides in a cubic symmetry is associated with a complex modification of the oxygen sub-lattice, which cannot be properly discriminated. Additionally, one of the fundamental postulates used for phase diagram assessment is that U_4_O_9_, U_3_O_7_ and U_3_O_8_ are stoichiometric compounds while non-stoichiometry may exist in these oxide phases. On the other hand, XANES probes directly the valence state (unfilled 6*d* and *5f* shells) of the cation through 2*p*–6*d* transitions (*L*_II,III_ edge) and the associated oxygen stoichiometry is derived applying the electroneutrality rule. This technique appears then as a method of choice as illustrated by its application at room temperature for lanthanides- and actinides-doped UO_2_ compounds (Prieur *et al.*, 2011 ▸, 2013 ▸, 2018*a* ▸; Martin *et al.*, 2003 ▸). Nevertheless, *in situ* XANES application to U oxides remains extremely rare and has been limited to the UO_2_–UO_2+*x*_ domain (Prieur *et al.*, 2018*b* ▸; Caisso *et al.*, 2015 ▸). In this context, the second goal of this paper is to show the relevance of *in situ* XANES for such phase diagram determination. In this framework, new experimental data points have been collected using this method and discussed in regard to the available data. Furthermore, thermodynamic calculations have been performed using the CALPHAD (CALculation of PHAse Diagram) method permitting to conclude about the relevance of those XANES-derived results.

## 2. Experimental methods

### 2.1. *In situ* XANES

*In situ* XANES measurements were conducted on square (1.5 mm × 1.5 mm) samples extracted from a 0.5 mm-thick disk cut from a UO_2_ dense pellet (98% of the theoretical density) sintered in Ar-4%H_2_ at 2023 K during 4 h. The surface exposed to the X-ray beam was polished up to a diamond finish and the samples were then annealed for 4 h under a dry reducing atmosphere (Ar-5%H_2_) at 1673 K in order to remove damage induced by polishing and to guarantee an O/U ratio equal to 2.00.

**Figure 2:**
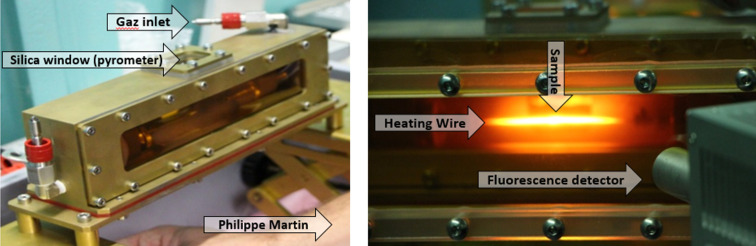
Photographs of the heating setup.

Prior measurement, the sample is mounted on a 1 mm-diameter Pt/Ir (90/10) wire of the furnace and embedded in a Pt/Ir (90/10) foil with a 0.5 mm hole allowing the incoming X-rays to hit the sample polished surface. Additional details on the sample positioning and a complete review of the heating wire have been given by Prieur *et al.* (2018*b* ▸) and Neuville *et al.* (2014 ▸), respectively. This heating element is then inserted into a dedicated furnace (Fig. 2 ▸) which allows collecting *in situ* XAS data on radioactive samples in various atmospheres and up to 2000 K. The Pt/Ir wire temperature was calibrated before the measurement. This heating system has low thermal inertia and it is possible to change the temperature from room temperature up to 2000 K and the inverse as well in a few seconds.

During the measurements, a constant gas flow of 8 L h^−1^ was maintained using a Bronkhorst numeric gas flow meter. The oxygen partial pressure in flowing gas was monitored by 1.2 bar mixing of Ar-4%H_2_, Ar, Ar-100 p.p.m. O_2_ and 80%N_2_–20%O_2_ gas bottles. The oxygen partial pressure was continuously measured at the entrance of the furnace using a Jok’air 2060 device (SETNAG company). This equipment can measure *p*(O_2_) from 10^−35^ to 0.25 atm, and the provider indicates a relative uncertainty of 3% for this entire range; however, according to the repeatability of our experiences, higher uncertainties up to 20% should be considered for *p*(O_2_) < 10^−6^ atm.

The *in situ* XANES measurements were conducted at the INE beamline of the KIT synchrotron light source (Karlsruhe Institute of Technology, Germany). The storage ring operating conditions were 2.5 GeV and 100–160 mA. A Ge [422] double-crystal monochromator coupled with collimating and focusing Rh-coated mirrors was used. XANES spectra were collected in fluorescence mode at the U *L*_III_ edge (17166 eV) with a single-element Si solid-state detector (Vortex 60EX, Hitachi, USA). Energy calibration was achieved by measuring the *K* XANES spectrum of a Y reference foil (17038 eV) located between the second and third ionization chambers. The XANES spectra have been normalized using linear functions for pre- and post-edge modelling. The white-line maxima have been taken as the first zero-crossing of the first derivative. Pre-edge removal, normalization and self-absorption correction were performed using the *ATHENA* software (Ravel & Newville, 2005 ▸). An example of self-aborption correction is provided in Figure S1 of the supporting information. The molar fractions of U^IV^, U^V^ and U^VI^ were derived from the linear combination fitting (LCF) of stoichiometric UO_2.00_, U_4_O_9_ and U_3_O_8_ references (Prieur *et al.*, 2011 ▸). Note that this fitting procedure is not affected by the temperature. The XANES region is indeed quite insensitive to the thermal disorder, as it notably exhibits a high signal-to-noise ratio (Prieur *et al.*, 2018*b* ▸).

### 2.2. Thermodynamical modelling

The description of multicomponent systems is based on the assessments of mainly binary and ternary subsystems using semi-empirical models to describe the thermodynamic properties of the stable phases. These models permit to describe Gibbs energies as a function of temperature, composition and pressure in the CALPHAD approach (Cacciamani, 2016 ▸; Kattner, 2016 ▸). In order to obtain the best fit of the available experimental data (phase diagram points, oxygen potential, enthalpy, melting point,…), adjustable parameters are optimized. The thermodynamic calculations have been performed with the *Thermo-Calc* (Kattner, 2016 ▸; Sundman *et al.*, 1985 ▸) software using the model derived by Guéneau *et al.* (2011 ▸), used in the TAF-ID database (Thermodynamic for Advanced Fuel – International Database Release 11).

We calculated the binary phase diagram and the oxygen potential evolution as a function of the O/U ratio for each test temperature using this model.

### 2.3. Gibbs energy model

In the CALPHAD method, the thermodynamic equilibrium is calculated by minimizing the total Gibbs energy of the system, which is a linear combination of each Gibbs energy phase function present in the system. These functions are described using the ‘sub-lattice model’ proposed by Guéneau *et al.* (2011 ▸) for the uranium–oxygen system. In this approach, the crystal structure of each phase known is decomposed in several sub-lattices and each one includes the different ionic species. Their relative different content is adjusted in order to respect the electroneutrality. The fluorite UO_2±*x*_ structure can hence be described as follows,

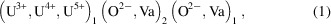

where ‘Va’ corresponds to the oxygen vacancies and the indexes 1 and 2 describe the stoichiometry of the compound.

As illustrated by the relation (1), the model is composed of one cationic sub-lattice and two anionic sub-lattices. The first is for the oxygen atoms on tetrahedral sites (the ‘normal’ O positions in stoichiometric UO_2_) and the second one for oxygen atoms in the interstitial position. Thanks to the sub-lattice model, the compound Gibbs energy is determined for each phase. All the Gibbs energy functions refer to the Stable Element Reference (SER) corresponding to the enthalpy of the pure elements in their standard state conditions (298.15 K and 10^5^ Pa) and they depend on the state variables such as temperature, composition and pressure leading to the general equation (2) for a pure element,

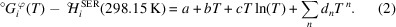

Furthermore, the Gibbs energy function for non-stoichiometric phases is written as the composition of different contributions, as follows,

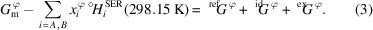

In this function, ^ref^ *G* ^φ^ corresponds to the Gibbs energy of the reference state, ^id^ *G* ^φ^ to the ideal random mixing contribution and ^ex^ *G* ^φ^ to the excess of Gibbs energy. Concerning the ideal Gibbs energy, it depends on interaction parameters between species *A* and *B*, noted 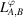 . Those parameters are expressed with the Redlich–Kister polynomial function in order to describe more precisely all the experimental data (Redlich & Kister, 1948 ▸). All the Gibbs energy functions for each phase of the U–O system used in the TAF-ID are detailed in the assessment by Guéneau *et al.* (2011 ▸).

### 2.4. Selection of oxygen potential data

At equilibrium, the oxygen potential of the solid sample is equal to the oxygen potential of the surrounding gas phase. It can be defined as

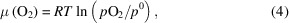

with *p*O_2_ the partial pressure of oxygen, *p* ^0^ the standard pressure (1 bar), *R* the gas constant and *T* the temperature.

**Figure 3:**
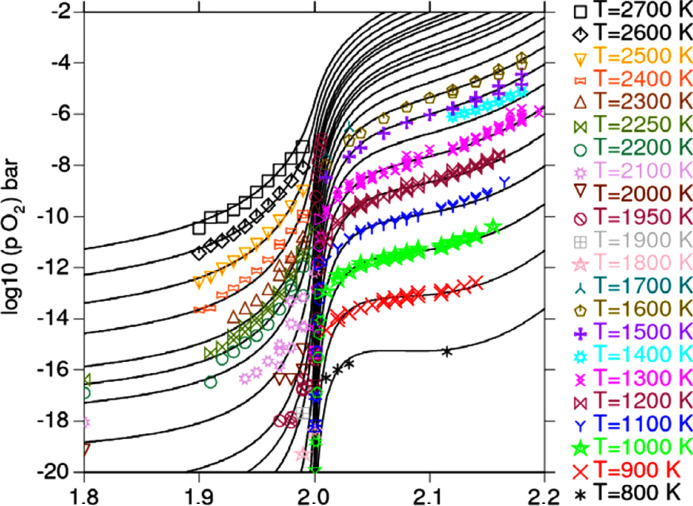
Oxygen activities in UO_2±*x*_ calculated (black line) with selected experimental data (coloured symbol) from Guéneau *et al.* (2011 ▸). Reprinted from Guéneau *et al.* (2011 ▸), Copyright (2011), with permission from Elsevier.

The different oxygen potential data sets have been critically selected by Labroche (Labroche, 2000 ▸; Labroche *et al.*, 2003*a* ▸,*b* ▸) and Baichi (Baichi, 2001 ▸; Baichi *et al.*, 2006*a* ▸,*b* ▸) and already used in the assessment of Guéneau *et al.* (2011 ▸). Those data correspond to the partial pressure or oxygen potential for different temperatures, for which the stoichiometry has been derived from various characterization methods (*e.g.* XRD, TGA,…). As illustrated by Fig. 3 ▸, showing the comparison between experimental and calculated oxygen activities in the UO_2±*x*_ domain, the evolution of the oxygen potential considerably varies with the O/*M* ratio and temperature. Furthermore, this representation shows a lack of experimental data for specific domains, according to the O/*M* ratio and the temperature. Experimental data are only available above 600 K, as shown in Fig. 3 ▸ (for a part of them).

The experimental thermodynamic data sets available in the literature corresponding to conditions used for *in situ* XANES measurements [298 (3), 448 (5), 773 (8), 1476 (15), 1483 (15), 1873 (19) and 1951 (20) K (a temperature range of ±120 K was considered)] are summarized in Table 1 ▸.

## 3. Results and discussion

**Figure 4:**
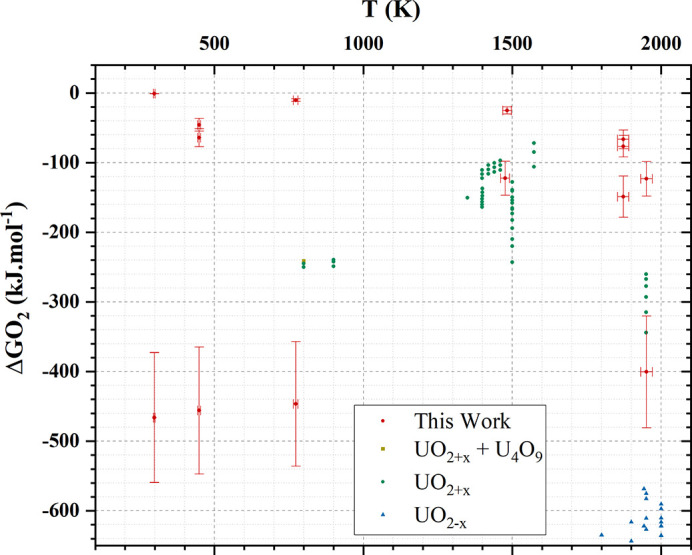
Oxygen potential (kJ mol^−1^) and temperature (K) of our experimental data and the experimental U–O thermodynamic data selected by Guéneau *et al.* (2011 ▸). Note that only bibliographic data corresponding to the range of our study have been plotted.

Fig. 4 ▸ presents the range of temperature and oxygen potential in which experimental data have already been collected. The yellow, blue and green symbols correspond to literature data which have been collected in different domains (UO_2_–U_4_O_9_, UO_2+*x*_ and UO_2–*x*_). Comparing with our new experimental points (red symbols), we observe that our study provides new experimental data and especially in condition domains which have not been studied before.

**Figure 5:**
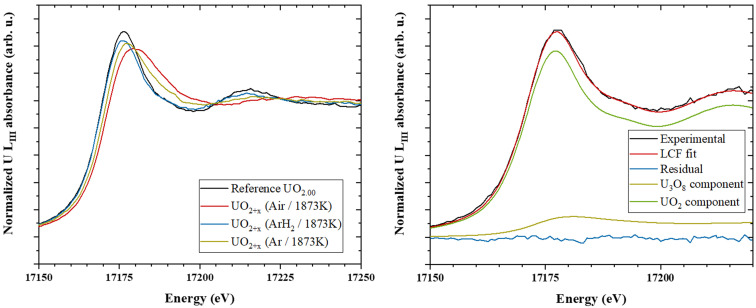
(*a*) U *L*_III_ XANES spectra of UO_2+*x*_ samples measured at 1873 (19) K in different oxygen potentials. (*b*) LCF of a U *L*_III_ XANES experimental spectrum fitted with UO_2_ and U_3_O_8_ components.

U *L*_III_ XANES spectra have been collected for each data point. Fig. 5 ▸(*a*) provides an example of XANES spectra recorded on a sample heated at 1873 (19) K in different atmospheres [*i.e.* −450 (90), −150 (30) and −50 (10) kJ mol^−1^]. The sample heated in the most reducing conditions (dry Ar-H_2_) is clearly stoichiometric because its white line is identical to the UO_2.00_ reference. On the contrary, a shift toward higher energy, as well as a broadening, appears when heated in more oxidizing conditions (*i.e.* Ar and air). These spectral changes indicate a modification of the U oxidation state, and especially of oxidation in the present case. Note that the variation of intensity between the UO_2.00_ reference and the experimental spectra is due to the increase of thermal vibrations.

The U *L*_III_ XANES spectra were fitted in order to determine the U valence and the corresponding molar fractions of each oxidation state. The basic principle of LCF is to fit the XANES experimental spectrum by combining XANES experimental spectra of reference materials. As an example, Fig. 5 ▸(*b*) shows a fit of an experimental spectrum using two components: UO_2_ and U_3_O_8_. The output of such procedure is the molar fractions of each component species, which allows deriving the molar fraction of U^IV^, U^V^ and U^VI^, as well as the O/U ratio.

**Figure 6:**
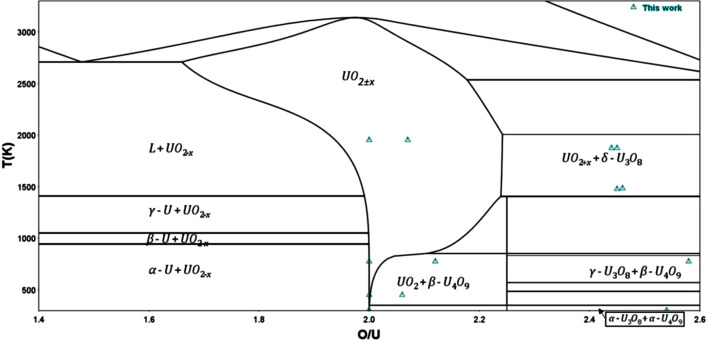
Our experimental point (blue triangles) in the calculated U–O phase diagram.

By plotting our O/U experimental values into the U–O phase diagram (Fig. 6 ▸), we can observe that our data are scattered in different domains: UO_2±*x*_, UO_2_–U_4_O_9_ and UO_2+*x*_–U_3_O_8_. It is remarkable to note that, for each collected experimental point, the best LCF results are systematically obtained using component species matching the end-members indicated in the phase diagram ( *cf.* Table S1 of the supporting information). For instance, the experimental spectrum of Fig. 5 ▸(*b*) has been fitted using UO_2_ and U_3_O_8_; and this experimental point is actually in the UO_2+*x*_–U_3_O_8_ domain of the U–O phase diagram. This supports the validity of the LCF approach to determine the O/U value.

**Figure 7:**
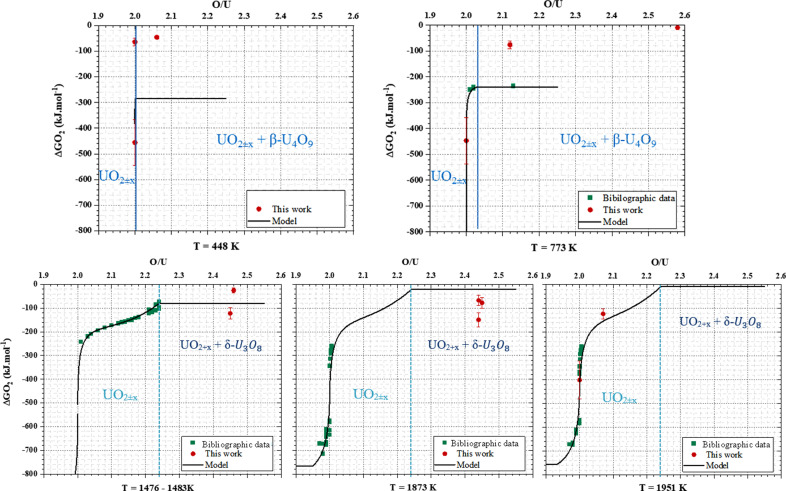
For *T* > 1400 K, comparison between the O/U calculated (black lines) and our experimental data (red circles), and the selected experimental data (green squares). Note that the oxygen potential curves (black lines) are solely based on the experimental data from the literature (green squares).

Fig. 7 ▸ compares, for a given temperature, our experimental values (red circles) with the oxygen potential curve (black lines) derived from the thermodynamic modelling. Note that the calculated data are extrapolated from the existing experimental data (green square) and the thermodynamic data of each U oxide end-members considered in the CALPHAD model.

Overall, we observe two main tendencies: for *T* > 1400 K, our data are in good agreement with the predicted values while a poor agreement is reached for *T* < 800 K. This corresponds actually quite well with domains in temperature and oxygen potential with a lack of experimental data points. In detail, our two experimental points at 1951 (20) K are in a very good agreement with the model. This can be understood from the fact that for this temperature the model is based on several experimental points ranging from O/U = 1.95 to O/*M* = 2.02 as illustrated by Table 1 ▸ and Fig. 7 ▸, which allows a proper extrapolation for higher O/U values. At 1876 (19) K, our experimental data correspond to a domain of oxygen potential where no data have been reported in the literature. The tendency is respected but the O/U values predicted by the model are much lower than the experimental data. For both 1476 (15) and 1483 (15) K, both experimental and calculated values are consistent. At lower temperature, *i.e.* 448 (5) and 773 (8) K, there is a perfect agreement for the stoichiometric values while experimental and calculated data do not match for higher O/U values. In that case, the kinetics of the reaction might play a role in the final O/U value but, undoubtedly, the main problem comes from the absence of experimental data in these conditions. According to the XANES, U(VI) is found solely in the biphasic domain while only U(IV) and U(V) are present in the UO_2+*x*_ monophasic domain. This is in agreement with the CALPHAD formalism which assumes that the UO_2+*x*_ structure is composed of U(IV) and U(V).

## 4. Conclusion

In this study, *in situ* XANES has been used to explore the U–O phase diagram and to collect new experimental data in condition domains in which experimental data were missing. *In situ* XANES is particularly relevant for such a purpose as, contrary to other methods, O/U can be determined independently of the crystallographic nature of the samples. Those new experimental results could be used to optimize the thermochemical model in the CALPHAD approach.

By itself, the *in situ* method already has a huge interest for a wide range of applications in which the oxidation states drive the chemical processes. Here we also demonstrated that *in situ* XANES coupled with thermodynamic calculations is a proper combination to assess phase diagrams. Indeed, *in situ* XANES allows collecting relevant data close to the real thermodynamic conditions encountered in the nuclear fuel cycle. More generally, this method can be employed for all U-based systems and could hence increase significantly the amount of experimental data to feed thermodynamical calculations.

Combining it with X-ray diffraction for example would of course unravel even more fruitful information, as one would be able to access both charge distribution and phase structure at the same time.

## Supplementary Material

Figure S1 and Table S1. DOI: 10.1107/S1600577521010572/yq5002sup1.pdf

## Figures and Tables

**Table 1:** Experimental U–O thermodynamic data selected in this present work

Data	References	Temperature domain
Data selected from Baichi *et al.* (Baichi *et al.*, 2006*a* ▸,*b* ▸; Baichi, 2001 ▸)		

Oxygen chemical potential in UO_2±*x*_	Tetenbaum & Hunt (1970 ▸), Pattoret (1969 ▸), Javed (1972 ▸), Markin & Bones (1962 ▸), Baker (1971 ▸), Wheeler (1971 ▸), Wheeler & Jones (1972 ▸)	2000 K
		2000 K
		1900–2000 K
		900 K
		1942 K
		1800–2000 K
		1950 K

Data selected from those of Labroche *et al.* (Labroche, 2000 ▸; Labroche *et al.*, 2003*a* ▸,*b* ▸)		

Oxygen chemical potential in UO_2±*x*_ and in UO_2–*x*_ + U_4_O_9_	Hagemark & Broli, (1966 ▸), Roberts & Walter (1961 ▸), Blackburn (1958 ▸), Markin & Bones (1962 ▸), Nakamura & Fujino (1987 ▸)	1500–1573 K
		1420–1500 K
		1399–1500 K
		773–900 K
		800–900 K
