# Supplementary material for: A spectroscopic hike in the U–O phase diagram
Source: J Synchrotron Radiat. 2021 Nov 3;28(Pt 6):1684–91. doi: 10.1107/S1600577521010572 (PMC8570218; doi:10.1107/S1600577521010572)
Supplement: Supplementary file 1 [file s-28-01684-sup1.pdf]

JOURNAL OF SYNCHROTRON RADIATION

Volume 28 (2021)

Supporting information for article:

A spectroscopic hike in the U–O phase diagram

Damien Prieur, Marie-Margaux Desagulier, Daniel R. Neuville, Christine Guéneau, Enrica Epifano, Kathy Dardenne, Joerg Rothe and Philippe Martin

Figure S1 Example of XANES data with and without self-absorption correction.

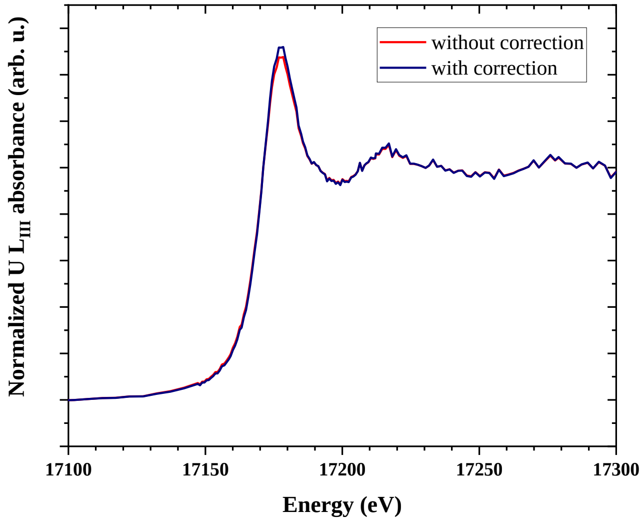

Table S1 Molar fractions and O/U derived from the LCF of the UL$_{III}$ XANES spectra.

T (K)	ΔGO_2 (kJ.mol^{-1})	UO_2 (%)	U_4O_9 (%)	U_3O_8 (%)	R factor (%)	U^{IV} (%)	U^{V} (%)	U^{VI} (%)	Valence U	O/U
298 (3)	-466 (90)	100 (5)	0 (5)	0 (5)	0.1	100 (5)			4.00	2.00 (1)
298 (3)	-466 (90)	100 (5)			0.5	100 (5)			4.00	2.00 (1)
298 (3)	-466 (90)	100 (5)			0.1	100 (5)			4.00	2.00 (1)
298 (3)	-1 (10)		51 (5)	49 (5)	0.1	26 (5)	42 (5)	33 (5)	5.07	2.54 (1)
448 (5)	-456 (90)	100 (5)	0 (5)	0 (5)	0.2	100 (5)			4.00	2.00 (1)
448 (5)	-64 (10)	100 (5)	0 (5)	0 (5)	0.2	100 (5)			4.00	2.00 (1)
448 (5)	-46 (10)	76 (5)	24 (5)	0 (5)	0.1	88 (5)	12 (5)		4.12	2.06 (1)
773 (8)	-447 (90)	53 (5)	47 (5)		0.2	77 (5)	24 (5)		4.24	2.00 (1)
773 (8)	-76 (10)	51 (5)	49 (5)		0.4	76 (5)	25 (5)		4.25	2.12 (1)
773 (8)	-10 (10)		44 (5)	56 (5)	0.2	22 (5)	41 (5)	37 (5)	5.15	2.58 (1)
1476 (15)	-122 (30)		65 (5)	35 (5)	0.3	33 (5)	44 (5)	23 (5)	4.91	2.45 (1)
1483 (15)	-25 (10)		64 (5)	36 (5)	0.1	32 (5)	44 (5)	24 (5)	4.92	2.46 (1)
1873 (19)	-149 (30)		67 (5)	33 (5)	0.2	34 (5)	45 (5)	22 (5)	4.89	2.44 (1)
1873 (19)	-77 (20)		66 (5)	34 (5)	0.1	33 (5)	44 (5)	23 (5)	4.90	2.45 (1)
1873 (19)	-67 (20)		67 (5)	33 (5)	0.2	34 (5)	45 (5)	22 (5)	4.89	2.44 (1)
1951 (20)	-401 (80)	100 (5)		0 (5)	0.4	100 (5)			4.00	2.00 (1)
1951 (20)	-123 (20)	72 (5)	28 (5)	0 (5)	0.2	86 (5)	14 (5)		4.14	2.07 (1)
